# Comparative Small RNA Analysis of Pollen Development in Autotetraploid and Diploid Rice

**DOI:** 10.3390/ijms17040499

**Published:** 2016-04-12

**Authors:** Xiang Li, Muhammad Qasim Shahid, Jinwen Wu, Lan Wang, Xiangdong Liu, Yonggen Lu

**Affiliations:** State Key Laboratory for Conservation and Utilization of Subtropical Agro-Bioresources, South China Agricultural University, Guangzhou 510642, China; andyslee@126.com (X.L.); qasim@scau.edu.cn (M.Q.S.); wjw0106@126.com (J.W.); wanglan@scau.edu.cn (L.W.)

**Keywords:** meiosis, microRNAs, polyploidy, pre-meiotic interphase (PMA), single microspore stage (SCP), siRNAs

## Abstract

MicroRNAs (miRNAs) play key roles in plant reproduction. However, knowledge on microRNAome analysis in autotetraploid rice is rather limited. Here, high-throughput sequencing technology was employed to analyze miRNAomes during pollen development in diploid and polyploid rice. A total of 172 differentially expressed miRNAs (DEM) were detected in autotetraploid rice compared to its diploid counterpart, and 57 miRNAs were specifically expressed in autotetraploid rice. Of the 172 DEM, 115 and 61 miRNAs exhibited up- and down-regulation, respectively. Gene Ontology analysis on the targets of up-regulated DEM showed that they were enriched in transport and membrane in pre-meiotic interphase, reproduction in meiosis, and nucleotide binding in single microspore stage. *osa-miR5788* and *osa-miR1432-5p_R+1* were up-regulated in meiosis and their targets revealed interaction with the meiosis-related genes, suggesting that they may involve in the genes regulation associated with the chromosome behavior. Abundant 24 nt siRNAs associated with transposable elements were found in autotetraploid rice during pollen development; however, they significantly declined in diploid rice, suggesting that 24 nt siRNAs may play a role in pollen development. These findings provide a foundation for understanding the effect of polyploidy on small RNA expression patterns during pollen development that cause pollen sterility in autotetraploid rice.

## 1. Introduction

The evolutionary history of all angiosperms shows that about 30%–60% of them might be polyploids, whole genome duplication has occurred in 75% of all flowering plants [1,2]. Polyploids could be an important source for plant breeders in the future [3,4] because it offers the following three advantages: increasing the variation in dosage-regulated gene expression evolved to new biological functions, advancing the largest vegetative organs, and high levels of heterosis by polyploidy hybrids, over diploid progenitors [5,6,7,8].

Autotetraploid rice is a useful germplasm resource obtained by chromosome doubling with strong hybrid vigor, ability of resistance against abiotic and biotic stresses, and high biomass production compared with diploid rice [9,10]. However, the poor seed set is the main barrier in commercial utilization of polyploid rice. Pollen sterility is one of the major factors that cause low seed set in polyploid rice [11,12,13]. Previous studies on autotetraploid rice indicated that microtubule organization and abnormal chromosome behavior may result in sterile pollens [11,12]. Microarray analysis revealed that the differential gene expressions altered the meiosis gene network that may result in abnormal chromosome behavior and pollen development in autotetraploid rice [14]. Recently, we conducted a transcriptomic analysis on allelic interactions in autotetraploid rice hybrids, and the results revealed that polyploidy enhanced F_1_ pollen sterility loci interactions that alter the expression profiles of meiosis-related or meiosis-stage-specific genes, and resulted in low pollen fertility in autotetraploid rice [15]. Though many studies have been conducted to understand the differences between autotetraploid and diploid rice at cytological and molecular levels, microRNAome analysis on developing pollens of autotetraploid rice is still poorly understood.

MicroRNAs (miRNAs) are small (20–25 nucleotides) single stranded non-coding RNAs that modulate plant gene expression by gene silencing through inhibition of target mRNAs. A number of studies have confirmed that miRNAs involved in a series of biological functions, plant growth, reproduction and responses to hormones and stresses [16,17,18,19]. Some pollen-specific miRNAs have been detected after uninucleate microspores stage of developing pollen in rice [20,21,22]. However, little is known about the changes in expression patterns of miRNAs and their association with pollen development in autotetraploid rice compared to diploid counterpart. Therefore, we planned this study to investigate the differentially expressed miRNAs between autotetraploid and diploid rice, and to detect novel miRNAs that may be associated with meiosis or other stages during pollen development in autotetraploid rice. The results of the present study will provide insights into the roles of miRNAs during pollen development in autotetraploid rice and their association with pollen fertility.

## 2. Results

### 2.1. Overview of MicroRNAs (miRNAs) Sequencing Datasets in Developing Pollens of Rice

To investigate the miRNAs associated with the pollen development of diploid (CK) and autotetraploid rice, three pollen development stages, PMA (pre-meiotic interphase), MA (meiosis) and SCP (single microspore stage), were selected to construct six libraries and sequenced by Solexa high-throughput sequencing technology. A total of 9,176,755, 9,017,836 and 9,271,861 at PMA, MA and SCP raw reads were obtained in each library of Taichung65-4x, respectively. After removing the low quality sequences, adaptor sequences and RNAs smaller than 18 nt (ACGT101-miR program), we obtained 5,841,382 (63.65%), 5,781,692 (64.11%) and 4,341,659 (46.83%) high quality clean reads, which represented 1,201,066 (53.59%), 1,603,531 (57.64%) and 1,792,394 (55.3%) unique sequences at PMA, MA and SCP libraries ranging from 18 to 30 nt in length in autotetraploid rice, respectively (Table S1). We detected 6,284,253 (70.52%), 6,903,329 (60.76%) and 4,008,705 (34.76%) clean reads that represented 1,362,318 (56.74%), 1,219,793 (55.03%) and 1,191,600 (48.57%) unique reads in Taichung65-2x at PMA-2x, MA-2x and SCP-2x, respectively (Table S1). These high-quality reads were mapped to rice precursors in miRBase to identify known and novel miRNAs for further analysis. Low level of large fragments, such as mRNA (messenger RNA) and rRNA (ribosomal RNA), were also found, which indicated the high-quality and no degradation of RNA samples in the present study. A total of 93.19% and 91.44% small RNAs identified here spanned a size range of 20–24 nt in Taichung65-4x and Taichung65-2x, respectively. After removing the redundant sequences, we found 24 nt sRNA sequences more frequently compared to other sRNAs sequences, and the second highest peak was observed for 21 nt sRNA sequences (Figure S1).

A total of 2336 miRNAs, classified into five categories (Table S2), were detected via BLAST in miRBase. After removing the less expressed miRNAs, *i.e.*, the expression levels were less than 10 after the normalization of dataset, we identified 486 miRNAs including 192 known miRNAs belonging to 112 families and 294 novel miRNAs during pollen development. Of these, 486 miRNAs, 441 and 402 were preferentially expressed in autotetraploid and diploid rice, respectively (Tables S3 and S4, respectively).

**Figure 1:**
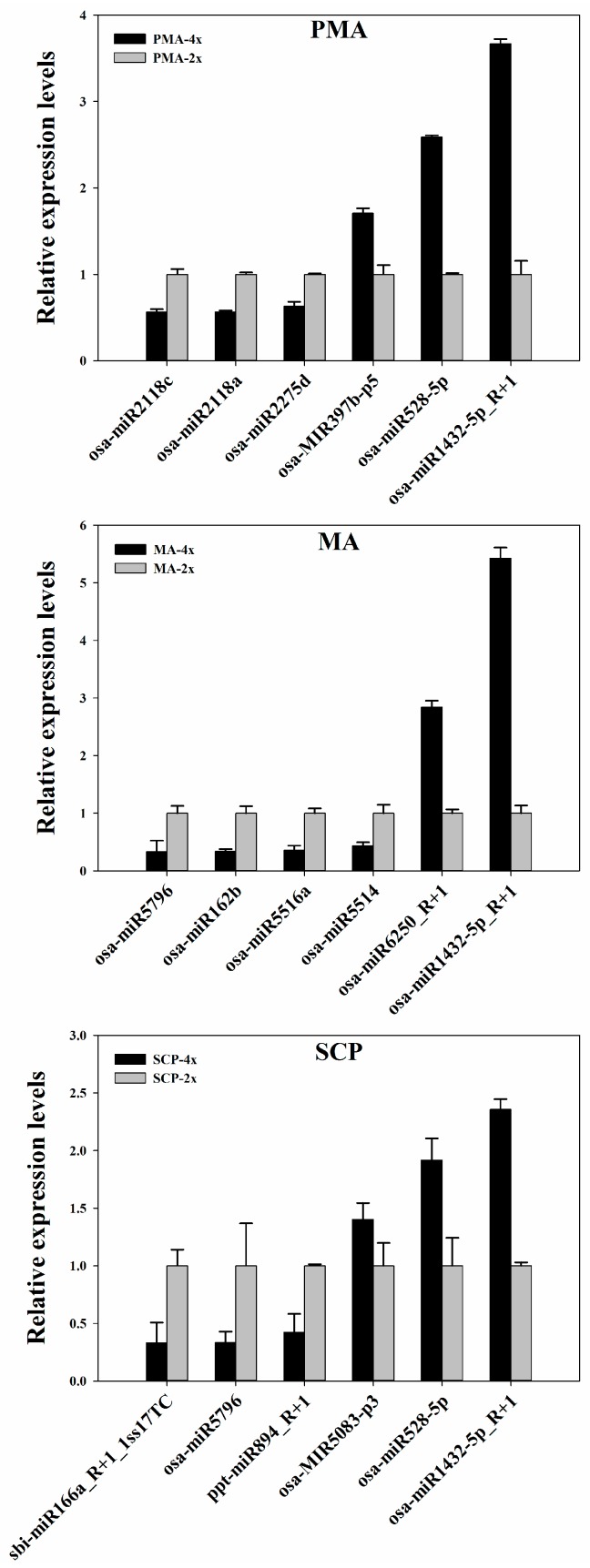
Validation of the DEM (differentially expressed miRNAs) in autotetraploid and diploid rice at each pollen development stage (pre-meiotic interphase (PMA), meiosis (MA) and single microspore stage (SCP)). *U6* snRNA was used as an internal reference for the qRT-PCR. The *x*- and *y*-axis represent the miRNAs and relative expression levels, respectively. Error bars represent the standard deviation (SD) of three biological replicates. “4x” and “2x” represent the autotetraploid and diploid rice, respectively.

The expression profiles of miRNAs detected in Taichung65-4x and Taichung65-2x during pollen development were compared using principal component analysis (Figure S2). The six samples were grouped into two categories, and the pollen development stages (*i.e.*, PMA, MA and SCP) could be clearly distinguished (Figure S3). These results demonstrated the reliability of the samples used in the present study. Six miRNAs were randomly selected from three pollen development stages for validating the differentially expressed miRNAs (DEM) by quantitative real-time PCR (qRT-PCR). The qRT-PCR results showed that their expression patterns were similar to those by the high-throughput sequencing, demonstrating the accuracy of small RNA sequencing results in the present study (Figure 1).

### 2.2. Association between miRNAs Expression Profiles and Pollen Development Stages in Autotetraploid and Diploid Rice

**Figure 2:**
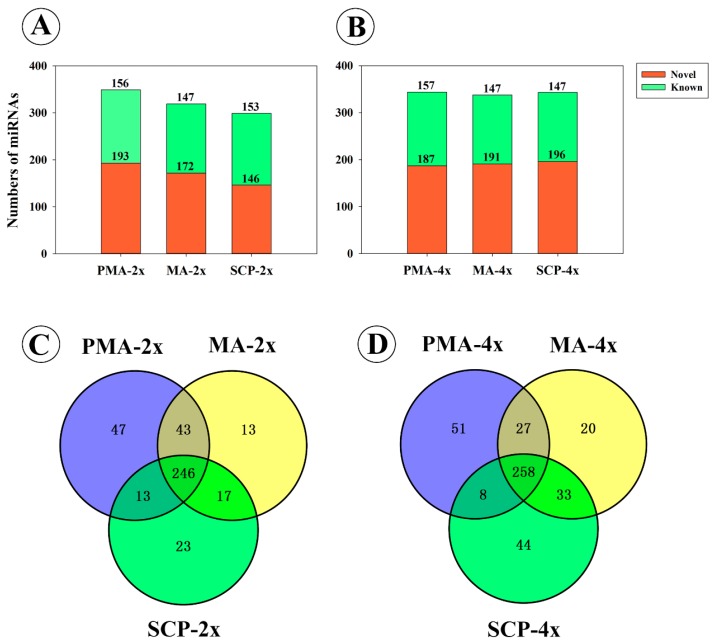

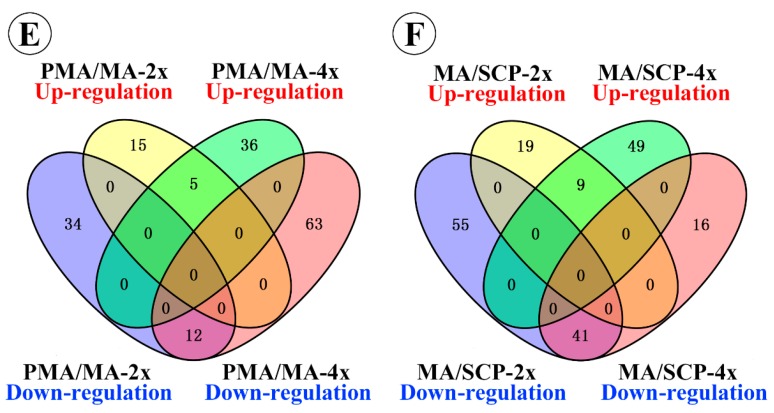
Classification of miRNAs during pollen development: (**A**,**B**) the total number of miRNAs detected during different pollen development stages of Taichung65-2x (**A**) and Taichung65-4x (**B**); (**C**,**D**) Venn analysis of miRNAs expressed in Taichung65-2x (**C**) and Taichung65-4x during pollen development (**D**); and (**E**,**F**) specifically up- and down-regulated miRNAs during each adjacent stage of pollen development in Taichung65-2x and Taichung65-4x. PMA, MA and SCP represent pre-meiotic interphase, meiosis and single microspore stage, respectively. “4x” and “2x” represent the autotetraploid and diploid rice, respectively.

A total of 402 miRNAs (*i.e.*, 349, 319 and 299 at PMA, MA and SCP, respectively), including 178 known and 224 novel miRNAs, were obtained during pollen development of Taichung65-2x by a threshold of >10 (Figure 2A; Table S3). Among 402 miRNAs, 246 miRNAs were co-expressed during the three pollen development stages (*i.e.*, PMA, MA and SCP) (Figure 2C), and 170 miRNAs showed stage-specific differential expression patterns during pollen development stages in the diploid rice (Table S5). Of the 170 differentially expressed miRNAs, 20 and 46 miRNAs in MA compared to PMA stage, and 28 and 96 miRNAs in SCP compared to MA stage were up- and down-regulated, respectively (Table 1 and Table S5). Of the 153 known miRNAs in SCP, 33 miRNAs displayed similar expression patterns as reported by Wei *et al.* [22], while 120 miRNAs were only found in SCP of Taichung65-2x (Table S6A). Of the 33 miRNAs, 30 miRNAs that belong to 21 miRNAs families were expressed in all development stages (Table S6B), and most of them were pollen-specific miRNAs.

A total of 441 miRNAs, including 173 known and 268 novel miRNAs, were detected in Taichung65-4x; of these, 344, 338 and 343 were identified in PMA, MA and SCP by a threshold of >10, respectively (Figure 2B; Table S4). Among 441 miRNAs, 258 miRNAs were co-expressed, and 208 miRNAs were stage-specific and exhibited differential patterns in pollen development stages (*i.e.*, PMA, MA and SCP) in autotetraploid rice (Figure 2D; Table S5). In total, 41 and 75 miRNAs in MA compared to PMA stage, and 58 and 57 miRNAs in SCP compared to MA stage were up- and down-regulated, respectively (Table 1 and Table S5). We also detected 32 known miRNAs in SCP of Taichung65-4x that were reported by Wei *et al.* [22] (Table S6A), and 29 of these 32 miRNAs were also expressed during the PMA and MA in autotetraploid rice (Table S6B). These 29 known miRNAs were detected in diploid rice as well, but some of them were found to be differentially regulated in both types of rice.

### 2.3. Differentially Expressed miRNAs during Pollen Development in Autotetraploid and Diploid Rice

**Figure 3:**
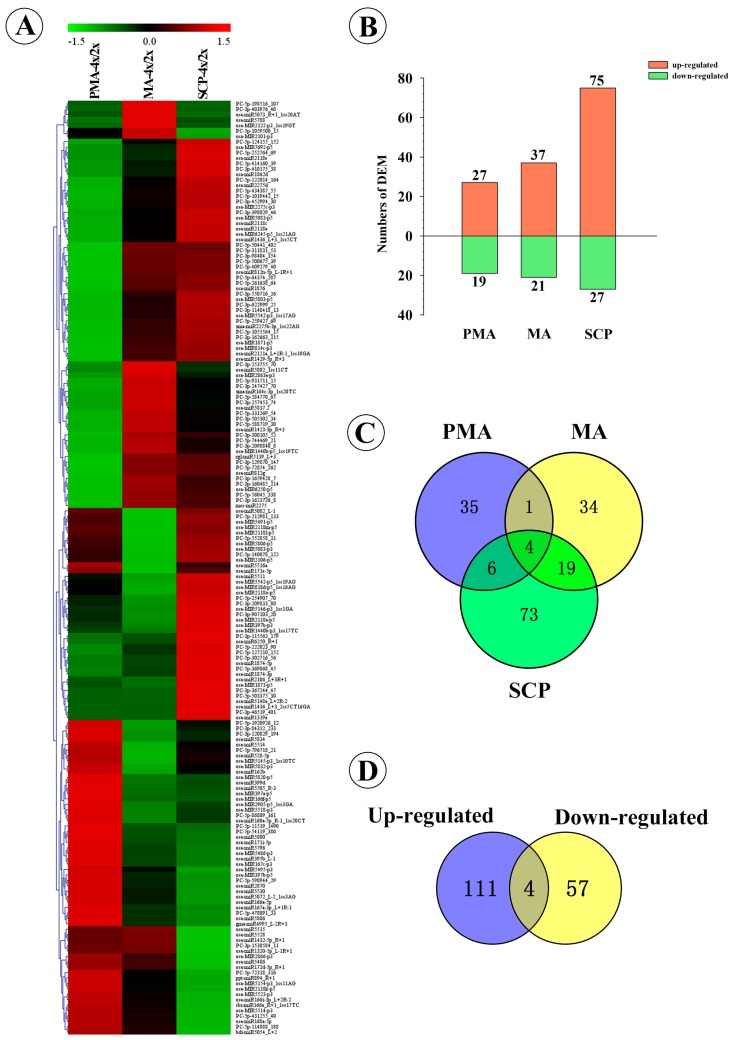
Analysis of DEM (differentially expressed miRNAs) in Taichung65-2x and Taichung65-4x during pollen development. (**A**) Hierarchical cluster analysis of DEM. The hierarchical clustering tree of 172 DEM in different libraries of pollen development was constructed by MultiExperiment View (version 4.9). Each column represents the difference between Taichung65-2x and Taichung65-4x in each stage. Red and green represent the up- and down-regulated miRNAs, respectively. The scale bar indicates the relative expression levels of miRNAs (log_2_); (**B**) The number of DEM at different pollen development stages; (**C**,**D**) Venn analysis of DEM during pollen development. PMA, MA and SCP represent pre-meiotic interphase, meiosis and single microspore stage, respectively. “4x” and “2x” represent the autotetraploid and diploid rice, respectively.

A total of 172 differentially expressed miRNAs (DEM), including 60 known and 112 novel miRNAs, which accounted for 35.39% of the total detected miRNAs, were detected during pollen development between autotetraploid and diploid rice (Figure 3A; Table S7). Among the DEM, 46 miRNAs were differentially expressed in PMA of Taichung65-4x compared with Taichung65-2x, of these 27 and 19 miRNAs were found to be up- and down-regulated, respectively. Fifty-eight miRNAs, including 37 up-regulated and 21 down-regulated, were differentially expressed in MA between Taichung65-4x and Taichung65-2x. One hundred two miRNAs were differentially expressed in SCP, among them 75 and 27 were up- and down-regulated, respectively (Figure 3B; Table 1). The minimum number of DEM was found in PMA and accumulated to maximum in SCP, consistent with the results of principal component analysis (Figure S2). A total of 142 miRNAs were specific/differentially expressed in the three pollen development stages (*i.e.*, 35, 34 and 73 in PMA, MA and SCP, respectively), which accounted for 82.56% of the DEM, and only four miRNAs (*osa-MIR5083-p5*, *osa-miR6250_R+1*, *osa-miR1320-5p_L-1R+1* and *osa-miR1432-5p_R+1*) were found to be differentially co-up-regulated in all three pollen development stages (Figure 3C; Table S7). We summed up the differentially expressed miRNAs in each stage, and 111 miRNAs showed up-regulation and 57 revealed down-regulation in autotetraploid rice (Figure 3D). However, we detected four miRNAs that showed the reverse tendency of regulation in different stages (Figure 3D), including *osa-MIR1871-p5*, *osa-miR1429-5p_R+3*, *osa-MIR2275c-p3* and *osa-miR2275d*, which were down-regulated in PMA but up-regulated in SCP (Table S7).

A total of 223 miRNAs were co-expressed in diploid and autotetraploid rice, some of them, such as *miR159*, *miR166*, *miR396*, *miR2118* and *miR2275* were highly expressed in both types of rice, indicating their conserved and essential roles in pollen development. Moreover, we identified 38 and 57 miRNAs, specifically associated with diploid and autotetraploid rice, respectively. Of these, 21, 7 and 10 miRNAs were expressed in diploid rice at PMA, MA and SCP, while 16, 13 and 28 were expressed during the above mentioned pollen development stages in autotetraploid rice, respectively (Table S8).

We have detected a large number of stage-specific differentially expressed miRNAs from PMA to SCP in autotetraploid rice compared to diploid rice. Among the stage-specific DEM, 36 up- and 63 down-regulated stage-specific DEM were detected from PMA to MA in autotetraploid rice compared to diploid, whereas only 5 up- and 12 down-regulated stage-specific DEM were common in the two lines (Figure 2E; Table 1). We detected 49 up- and 16 down-regulated stage-specific DEM that were specifically found in SCP compared to MA in autotetraploid rice, while 19 up- and 55 down-regulated in diploid rice (Figure 2F; Table 1). A total of nine up- and 41 down-regulated stage-specific DEM were found to be common in the two types of rice (Figure 2F; Table 1). Of these co-expressed stage-specific DEM, novel miRNA *PC-5p-11519_1490* showed down-regulation in MA compared to PMA of autotetraploid rice, *osa-miR5511* and *osa-miR5516a* depicted down-regulation in SCP compared to MA in autotetraploid rice (Table S5). These miRNAs were also found to be differentially expressed in autotetraploid rice compared to diploid rice during PMA, SCP and MA (Table S7).

### 2.4. Target Prediction of Differentially Expressed miRNAs (DEM) and Functional Classification

Identifying regulatory mRNA targets is essential for illustrating the function of miRNAs. To understand the effect of mRNA targets associated with the genetic variation of pollen development in autotetraploid rice, the predicted targets of 172 DEM were analyzed. Of these, 108 DEM were found with 729 predicted targets (Table S9). We found that the 662 of 729 predicted targets were significantly (*p* ≤ 0.05) categorized into 15 Gene Ontology (GO) terms (Table S10). Among these terms, transport (GO: 0006810) and localization (GO: 0051179) were dominated in the main category of biological processes, and secondary metabolic process (GO: 0019748), signal transduction (GO: 0007165) and catabolic process (GO: 0009056) were also found in biological processes. In the molecular function category, three significant GO terms, including transporter activity (GO: 0005215), lipid binding (GO: 0008289) and receptor activity (GO: 0004872) were identified. Only one term, extracellular region (GO: 0005576) was significant in cellular component category (Table S10).

We found that 111 and 57 miRNAs showed up- and down-regulation in the three stages of pollen development in autotetraploid rice, respectively. Gene Ontology (GO) analysis of the predicted targets of 111 up-regulated DEM showed that they were significantly enriched in transport (GO: 0006810), catabolic process (GO: 0009056), transporter activity (GO: 0005215) and extracellular region (GO: 0005576) (Figure S4). The predicted targets of 57 down-regulated DEM were enriched in multicellular organismal development (GO: 0032501), reproduction (GO: 0000003), signal transduction (GO: 0007165), transcription regulator activity (GO: 0030528) and nucleus (GO: 0005634) (Figure S5).

GO enrichment categories were specifically constructed by the predicted targets of up-regulated DEM in each stage, such as transport (GO: 0006810) and membrane (GO: 0016020) in PMA (Figure S6); response to stimulus (GO: 0050896) and reproduction (GO: 0000003) in MA (Figure S7); and transferase activity (GO: 0016740) and nucleotide binding (GO: 0000166) in SCP (Figure S8). Interestingly, the analysis of the predicted targets of down-regulated DEM revealed the highest number of GO categories in SCP, including multicellular organismal development (GO: 0007275), signal transmission (GO: 0023060), nitrogen compound metabolic process (GO: 0006807), and lipid binding (GO: 0008289) (Figure S9), while nucleotide binding (GO: 0000166) and RNA binding (GO: 0003723) terms were preferentially enriched in PMA and MA, respectively (Figures S10 and S11).

### 2.5. Functional Classification of Meiosis-Related Predicted Targets of Differentially Expressed miRNAs

Meiosis is a key stage of pollen development in autotetraploid rice; hence, we analyzed the DEM at this stage. We compared the target genes that were predicted by the DEM in autotetraploid rice with the reported microarray data for meiosis-related and stage-specific expression in diploid and autotetraploid rice [14,23,24,25,26,27]. Using the predicted targets of the down-regulated miRNAs, we identified one meiosis-specific gene (*i.e.*, *LOC_Os09g39400* gene was predicted by *osa-MIR5486-p3* and annotated as histidine-containing phosphotransferase protein) (Table S11). Three meiosis-related genes were identified in this study, including *LOC_Os10g17489* (predicted by *osa-miR160a-5p_R-1_1ss20CT*), *LOC_Os11g03300* (predicted by *osa-MIR2118l-p5*), and *LOC_Os08g45160* (predicted by *osa-MIR5806-p5*), which encode UDP-glucoronosyltransferase and UDP-glucosyltransferase domain containing protein, NAC domain transcription factor, and TENA/THI-4 family protein, respectively. These meiosis-related genes showed up-regulation in autotetraploid compared to the diploid rice during meiosis [14]. Additionally, two meiosis-related genes, *LOC_Os10g40420* (predicted by *osa-miR5072_L-2_1ss3AG*) annotated as LTPL138-Protease inhibitor/seed storage/LTP family protein precursor and *LOC_Os07g22930* (predicted by *osa-miR160a-5p_R-1_1ss20CT*) that encodes starch synthase were also found in this study, which exhibited differential expression patterns in diploid rice during meiosis [24] (Table S11). Of the predicted targets of up-regulated miRNA during meiosis, we identified four genes, including *LOC_Os01g64100* (predicted by *osa-miR1320-5p_L-1R+1*) encoded glycosyl hydrolase, *LOC_Os11g02400* (predicted by *osa-miR5788*) annotated as LTPL8-Protease inhibitor/seed storage/LTP family protein precursor, *LOC_Os12g02330* (predicted by *osa-miR5788*) documented as LTPL13-Protease inhibitor/seed storage/LTP family protein precursor, and *LOC_Os08g39000* (predicted by *osa-MIR6250-p5*) annotated as expressed protein; and these four predicted target genes were up-regulated in autotetraploid compared to diploid rice [14] (Table S11). Moreover, *osa-MIR2122-p3_1ss19GT* targeted *LOC_Os05g33390* (cation-transporting ATPase, annotated as the MALE GAMETOGENESIS IMPAIRED ANTHERS gene in *Arabidopsis thaliana* [28]) and *LOC_Os04g58960* (regulator of chromosome condensation) associated with the meiosis and involved in pollen development in rice, was specifically up-regulated in MA (Table S9A).

**Figure 4:**
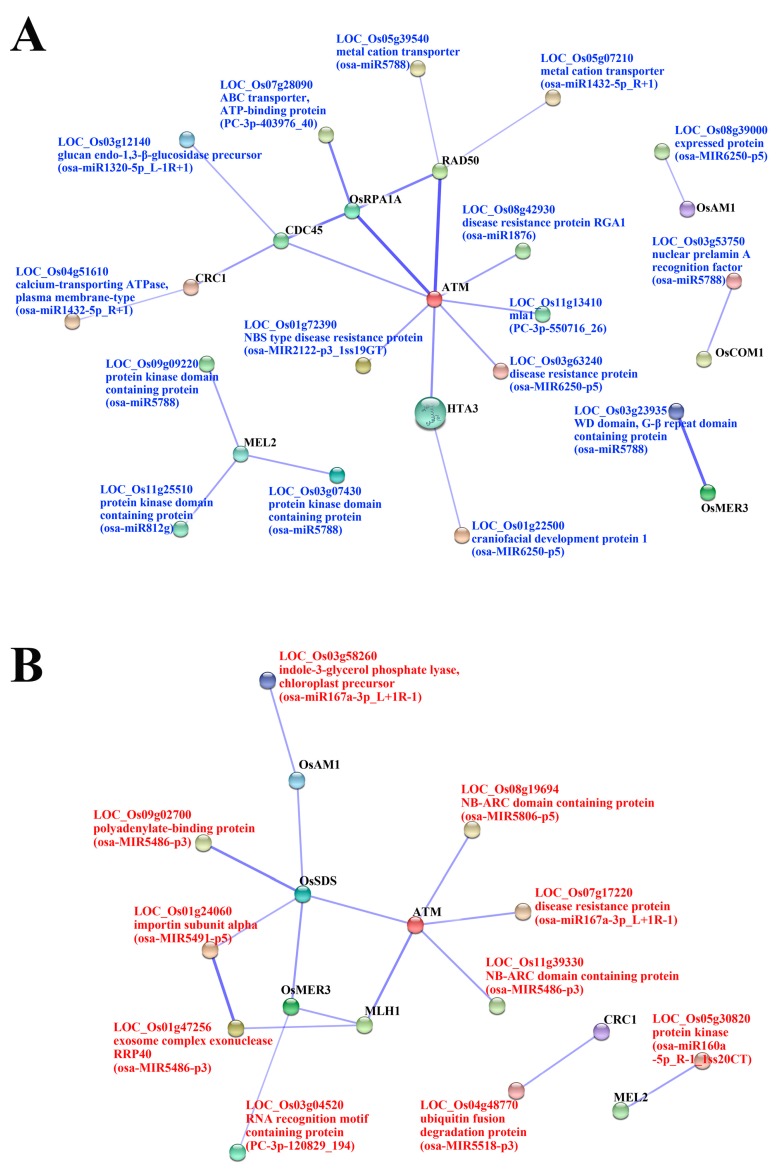
Protein interaction between the targets of DEM (differentially expressed miRNAs) and meiosis-related genes. The predicted targets of up-regulated (**A**) and down-regulated miRNAs (**B**) showed interaction with the meiosis-related genes (Black font). Blue font and red font represent the targets of up- and down-regulated DEM, respectively. Line thickness represents interaction between proteins/genes.

We identified 92 genes with known/proposed meiotic functions (Table S12) based on the putative meiosis-related genes detected by recent transcriptome analyses in rice and *Arabidopsis* [23,25,29,30]. Then we performed the protein–protein interactions of the important targets (detected at MA division) and 92 meiosis-related genes by using STRING v10. We identified 16 DEM that were associated with the meiosis (Figure 4; Table 2). Among the meiosis-related genes, HTA3, ATM, RAD50, CDC45, CRC1 and OsRPA1A were associated with the targets of up-DEM in MA stage, and constructed a meiosis-related gene-interaction network (Figure 4A). Three predicted targets of *osa-MIR6250-p5*, including *LOC_Os03g63240* (disease resistance protein), *LOC_Os01g22500* (craniofacial development protein 1) and *LOC_Os08g39000* (expressed protein), were associated with three meiosis-related genes (*i.e.*, ATM, HTA3 and OsAM1), respectively (Table 2). *LOC_Os05g07210* (predicted by *osa-miR1432-5p_R+1*) and *LOC_Os05g39540* (predicted by *osa-miR5788*) annotated as metal cation transporter, and showed interaction with RAD50 (Table 2). Additionally, *osa-miR5788* showed the interaction with four meiosis-related genes that were specifically up-regulated in MA of Taichung65-4x, and one of its targets, *LOC_Os03g23935* (*WD* domain, G-β repeat domain containing protein), showed interaction with *LOC_Os02g40450* (OsMER3) (Table 2). Novel miRNA, *osa-MIR2122-p3_1ss19GT* targeted of *LOC_Os01g72390* (NBS type disease resistance protein) that related to *LOC_Os01g01689* (ATM), and *PC-3p-403976_40* targeted of *LOC_Os07g28090* (ABC transporter, ATP-binding protein) that related to *LOC_Os02g53680* (OsRPA1A) were also up-regulated in MA (Table 2).

**Figure 5:**
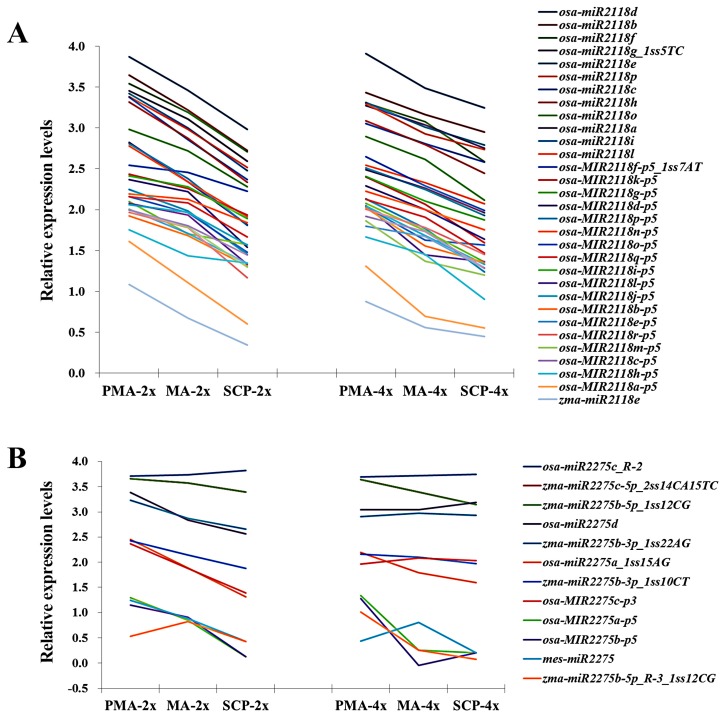
The relative expression levels of *miR2118* (**A**) and *miR2275* (**B**) families between Taichung65-4x and Taichung65-2x. PMA, MA and SCP represent pre-meiotic interphase, meiosis and single microspore stage, respectively. “4x” and “2x” represent the autotetraploid and diploid rice, respectively.

Furthermore, some miRNAs played key roles during meiotic division by generating phasiRNAs [31,32], such as *miR2118* and *miR2275* that required for biogenesis of phasiRNAs*.* The expression patterns of *miR2118* and *miR2275* families from PMA to SCP were almost the same between Taichung65-4x and Taichung65-2x, but with some variations in the members of these families (Figure 5). Eight miRNAs of *miR2118* family were differentially expressed and preferentially down-regulated in pollen development stages. Three of them, *osa-MIR2118l-p5*, *osa-MIR2118m-p5* and *osa-MIR2118o-p5*, were specifically down-regulated in MA. Four members of the *miR2275* family also exhibited differential expression patterns in Taichung65-4x. Interestingly, these miRNAs showed similar expression levels in MA, but significantly down-regulated in PMA (Table 3).

### 2.6. Functional Classification of Pre-Meiotic Interphase (PMA) and Single Microspore Stage (SCP) Stage-Specific Targets Regulated by Differentially Expressed miRNAs

Pre-meiotic interphase (PMA) is an important step for meiosis initiation, and many genes expressed during this stage. Consequently, the target genes predicted by differentially expressed miRNAs in autotetraploid rice were compared with the previously reported microarray data for pre-meiosis-related and stage-specific expression in diploid and autotetraploid rice during PMA [14,23,24]. Of the 27 up-regulated miRNAs in PMA, 22 predicted target genes were associated with PMA-related and stage-specific expression patterns (Table S11). One gene, *LOC_Os01g43870* (predicted by *PC-5p-431255_40*), encoded NLI interacting factor-like phosphatase, was found in this study, which showed down-regulation in PMA of autotetraploid compared to diploid rice in a previous study [14]. Eight predicted target genes were up-regulated in the same stage of autotetraploid than did in the diploid rice [14]. Another 18 genes were found to be up-regulated during the same stage in diploid rice [24]. Of the 19 down-regulated miRNAs in PMA, only one gene, *LOC_Os05g34220* (predicted by *osa-miR2118c*) was annotated as vrga1 and necessary for PMA in diploid rice, was specifically expressed in PMA (Table S11). In addition, *PC-5p-431255_40* was predicted to target the *LOC_Os02g07180* (JASON), *LOC_Os03g50220* and *LOC_Os06g51220* gene encoding an expressed protein, homologous-pairing protein and HMG1/2 (HIGH MOBILITY GROUP), which was specifically up-regulated in autotetraploid rice during PMA (Table S9A). These three genes were involved in male meiosis II [33], chiasma assembly [34] and chromatin assembly or disassembly [35] in *Arabidopsis*. Here, *osa-MIR167c-p3* targeted *LOC_Os05g31230* gene that encodes *N*-acetyltransferase, a homolog of the yeast CTF protein [36] that required for the formation of sister chromatid cohesion, which was also specifically up-regulated in autotetraploid rice during PMA (Table S9A).

Single microspore stage (SCP) development from meiosis owns to haploid chromosome, from which pollen development involves a highly coordinated series of cellular events; therefore, transcriptome is different from the somatic cells. We analyzed the DEM at this stage, and compared the target genes predicted by the DEM in autotetraploid rice with the previously known microarray data for diploid and autotetraploid SCP-related and stage-specific expression patterns [14,24]. Among 75 up-regulated miRNAs in SCP, 25 predicted target genes displayed stage-specific expression patterns. Of these 25 genes, *LOC_Os01g64100* (predicted by *osa-miR1320-5p_L-1R+1*) and *LOC_Os01g57610* (predicted by *osa-miR1436_L+3_1ss5CT*) exhibited up-regulation in SCP of autotetraploid compared to diploid rice [14]. Six predicted target genes of down-regulated miRNAs exhibited SCP-specific expression, and five of them were found to be up-regulated in SCP of diploid rice [24], and *LOC_Os01g03330* gene (predicted by *PC-5p-72318_316*) was up-regulated in SCP of autotetraploid compared to diploid rice [14] (Table S11).

### 2.7. Correlation between miRNAs and Their Target Genes

To validate the correlations between DEM identified by Illumina sequencing and their potential targets predicted by Targetfinder (MIT, Cambridge, MA, USA), five DEM and their corresponding target genes were taken during different pollen development stages in Taichung65-2x and Taichung65-4x, and examined by qRT-PCR (Figure S12). *osa-miR159a.1* targeted a *MYB* transcription factor family (*LOC_Os01g59660*), *osa-miR5514* and *osa-MIR5083-p3* targeted *LOC_Os01g49490* and *LOC_Os05g05440* gene, respectively. *osa-miR528-5p* targeted a *F-box* domain and *LRR* containing protein (*LOC_Os06g06050*) and *osa-miR1432-5p_R+1* targeted a metal cation transporter (*LOC_Os05g07210*). The expression patterns of DEM by qRT-PCR were nearly similar to the sequencing data during pollen development. The expression levels of three miRNAs were found to be negatively correlated with their target genes in Taichung65-2x compared to Taichung65-4x rice during SCP (Figure S12). In our study, *osa-miR528-5p* was up-regulated in PMA and SCP, while *osa-miR1432-5p_R+1* showed up-regulation during three pollen development stages in Taichung65-4x. However, *osa-miR528-5p* and *osa-miR1432-5p_R+1* showed negative correlation with their predicted targets in SCP. Different expression levels in Taichung65-4x may be due to the gene dosage effect of polyploidization.

### 2.8. Differentially Expressed siRNAs in the Autopolyploid Pollen Associated with Transposable Elements

**Figure 6:**
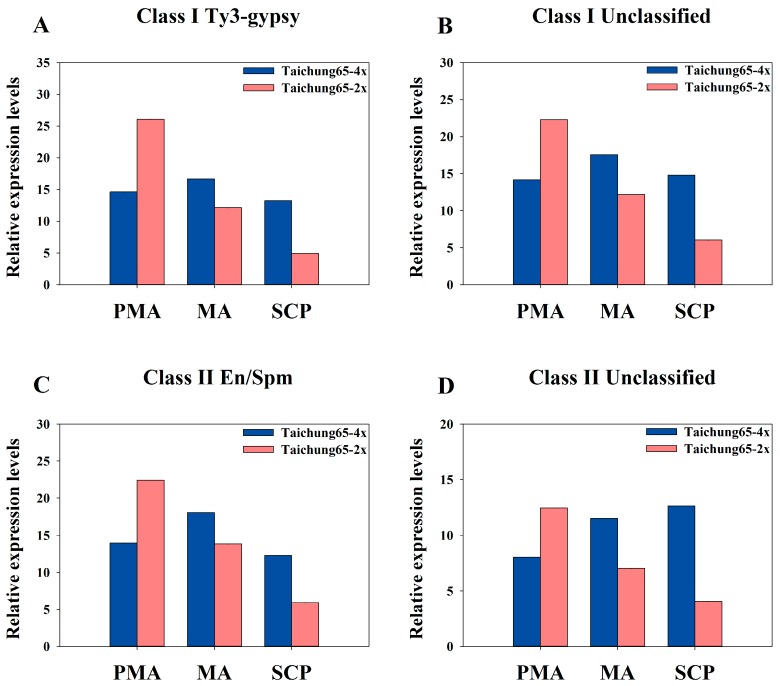
Abundance of siRNAs transposable elements associated with Taichung65-4x and Taichung65-2x during pollen development stages: (**A**) Ty3-gypsy type of class I; (**B**) unclassified type of class I; (**C**) En/Spm type of class II; and (**D**) unclassified type of class II. PMA, MA and SCP represent pre-meiotic interphase, meiosis and single microspore stage, respectively. “4x” and “2x” represent the autotetraploid and diploid rice, respectively.

Small RNAs regulates gene expression and directed DNA methylation [37]. The 24 nt siRNA abundance associated with CHG and CHH hypermethylation of class II transposable elements (TEs) inhibited the expression of related genes in autotetraploid rice [38]. Based on the method described by Zhang *et al.* [38], we identified a total of 1096 siRNAs associated with the transposable elements (Table S13); 21 to 24 nt sRNAs constituted the major portion of siRNAs population (Table S14). We focused on the 24 nt siRNAs (TEs) to investigate the relationship with DNA methylation in autotetraploid compared to diploid rice. We found five types of class I (retrotransposons) and five types of class II (transposon) in 24 nt siRNAs population; Ty3-gypsy and unclassified type with the number of 72 and 171 mainly related to class I (retrotransposons), whereas En/Spm and unclassified type with 84 and 38 in class II (transposon), respectively (Table S15). Thus, we mainly focused on these four main types of siRNAs. Compared to the diploid rice, 24 nt siRNA associated with transposable elements were more abundant during the pollen development in Taichung65-4x, except the PMA stage; very similar changes were detected in class I and class II (Figure 6). The siRNAs with lower expression levels, TEs associated, were found in PMA of polyploid than diploid rice. However, this tendency was reversed during MA and maintained in SCP of polyploid rice (Figure 6). In addition, the expression levels of TEs-associated siRNAs displayed remarkable changes in diploid rice, which gradually declined during the pollen development (Figure 6; Table S16). However, we found irregular changes in the expression levels of siRNAs during the pollen development in polyploid rice, and class I and class II of TEs were enriched in MA compared to PMA in polyploid rice (Figure 6; Table S16).

Furthermore, we compared the predicted targets of differentially expressed miRNAs detected in our study to Zhang *et al.* [38], and the results revealed that three targets associated with the DNA methylation activity, including *LOC_Os01g25450* (predicted by *mes-miR2275*, the down-regulated DEM in PMA), *LOC_Os10g26430* (predicted by *osa-miR5796*, the down-regulated DEM in MA and SCP) and *LOC_Os05g49930* (predicted by *bdi-miR5054_L+2*, the down-regulated DEM in SCP), were annotated as AIG1 family protein, agenet domain-containing protein and GRAS family transcription factor domain-containing protein, respectively (Table S17).

## 3. Discussion

### 3.1. Polyploidy Cause Changes in miRNA Expression Profiles during Pollen Development of Autotetraploid Rice

As negative regulators, miRNAs targets involved in signal transduction, carbohydrate and nitrogen metabolism and hormone homeostasis, indicating that miRNAs play important roles in regulating pollen development in plants [39,40,41] and rice grain filling [42,43]. For example, *miR156/7* targets *SPL* genes required for male fertility in *Arabidopsis* [44]. The *miR159* regulated the *GAMYB-*like targets (*MYB33* and *MYB65*) during *Arabidopsis* anther development [45]. The *miR167* controlled the expression patterns of *ARF6* and *ARF8* in *Arabidopsis* and regulate both male and female reproduction [46]. A recent study on miRNAome analysis of developing rice pollen from the uninucleate microspores (UNM) to tricellular pollen stages (TCP) revealed 202 known miRNAs in developing pollen, many (103) were pollen enriched, such as *osa-miR164a/b/f* and *d*, *osa-miR169e* and *n/o*, *osa-miR171a*, *osa-miR396a/b*, *osa-miR399h* and *osa-miR1881*, and more than half of 75 novel miRNAs were expressed in developing pollen [22]. Epigenetic regulation of chromatin assembly and disassembly might be controlled by the pollen development through over-expression of *osa-miR820* and *osa-miR827*, targeted *Os03g02010* (DNA cytosine methyl transferase) and *Os04g11510* (a methyl-CpG binding domain protein), respectively. Here, we detected significant differences in the abundance of miRNAs during the pollen development stages between autotetraploid and diploid rice; large numbers of specifically up- and down-regulated miRNAs were also found in the same adjacent stages between Taichung65-2x and Taichung65-4x, it may happen due to the effect of polyploidization. Additionally, a total of 223 miRNAs co-expressed in diploid and autotetraploid rice, while only 57 and 38 miRNAs were specifically expressed in autotetraploid and diploid rice, respectively. Autotetraploid rice inherited many miRNAs from the diploid rice; however, some were lost or generated after polyploidization. This phenomenon was also observed in other plants [47] and illustrated as the conserved miRNAs maintained genomic stability and the transcriptionally altered miRNAs might fulfill the need of polyploidy, such as the regulation of pollen fertility.

Furthermore, about 40% miRNAs (60 known miRNAs and 112 novel miRNAs) displayed different levels of expression during pollen development stages in autotetraploid and diploid rice. DEM were specifically found during each pollen development stage, suggesting a strong influence of miRNAs on their predicted target genes associated with the regulatory network of pollen development, and most probably resulted in the sterile pollen after the polyploidization. Autotetraploid rice has doubled genomes, more miRNAs should be observed and the expression levels should be increased two-fold compared with diploid rice. However, the expression patterns of miRNAs were not simplified between autotetraploid and diploid rice, some displayed over-expression while some showed down-regulation in autotetraploid rice than did in the diploid rice, as similar behavior was found during the miRNAome analysis in tetraploid *Paulownia tomentosa* [48]. Ha *et al.* [49] demonstrated that miRNAs expression levels are highly variable between the allotetraploids and their diploid counterpart (*Arabidopsis thaliana* and *Arabidopsis arenosa*), leading to the variations in gene expression, growth, and adaptation. Our data also suggest that miRNA regulation is more complex in autotetraploids than diploids.

GO analysis of the predicted targets of detected miRNAs in Taichung65-4x showed significant GO terms enriched in three stages, whereas some significant GO terms were detected only in one stage (Table S18). Our results showed that transcription factor activity was commonly enriched in three stages of Taichung65-4x. Similar phenomenon was found in the microarray analysis [14], indicating the importance of the transcription factor activity genes in developing pollens of autotetraploid rice. DEM predicted targets showed significant GO terms associated with the transport, transporter activity, signal and other growth and development processes. Among the DEM predicted targets, the predicted targets of up-regulated miRNAs were different from the down-regulated miRNAs. These results demonstrated that the differential expressions of miRNAs may be closely related to the pollen fertility of autotetraploid and diploid rice. In addition, a large number of targets involved in vegetative organ elongation and cell wall thickening were also found in our study. Recently, we have reported the larger PMC and the longer anther in autotetraploid than diploid rice [14], implying that the above mentioned genes have close relationship with cell wall structures of polyploidy.

### 3.2. Specific miRNA Expression Patterns May Cause Meiosis Abnormalities in Autotetraploid Rice

The 21- and 24-nucleotide phasiRNAs were generated by the target sites of *miR2118* and *miR2275*, recognized as a class of panicle-specific siRNAs [31,32]. Recently, rice Argonaute MEL1 selectively binds 21-nt phasiRNAs generated by *miR2118*, and the *mel1* mutants have abnormal cytoplasm and aberrant PMCs that halt in early meiosis, suggesting that MEL1-phasiRNAs play important role in male fertility [31,50]. In addition, *miR2118* family members were abundant at cell fate specification, and then vanished at cell differentiation of *maize* anther [32]. Similarly, our study also showed that *miR2118* was abundant in pre-meiotic and then gradually decreased during other stages of pollen development. MEL1 (*Os03g0800200*) was significantly up-regulated in Taichuang65-4x [14], closely related to the differential expressions of *miR2118*. Furthermore, the *miR2275* and meiotic-phasiRNAs accumulate preferentially in tapetum and meiocytes to perform their functions [32]. Lower accumulation of *miR2275* may cause developmental defects by disrupting the stamen in *osdcl4-1* [51]. In our study, the members of *miR2275* showed similar variation patterns in diploid rice, while some members displayed contrasting patterns from PMA to SCP, especially in meiosis of autotetraploid that probably give rise to unbalanced regulation of 24-phasiRNAs. These findings were associated with the higher frequency of cytological abnormalities in autotetraploid rice, and we inferred that differential expressions of *miR2118* and *miR2275* families were associated with meiotic abnormalities in autotetraploid rice.

By the protein–protein interaction analysis, we identified 16 meiosis-related miRNAs whose targets showed interaction with meiosis-related genes. One of predicted target, *PC-3p-403976_40*, was related to Replication Protein A (RPA1a). *OsRPA1a* is required for meiosis, the *osrpa1a* mutants were sterile at the reproductive stage and no embryo sac was found in female meiocytes, and defragmented chromosome were observed in male meiocytes after anaphase I [52]. *OsMER3* is necessary for normal meiotic crossing over, and null mutation of MER3 cause complete sterility in rice [53]; *OsMER3* was associated with a target predicted by *osa-miR5788* in our study. HTA3, ATM and RAD50 associated with each other in the protein interaction network. ATM gene, a chromosome instability disorder in *Arabidopsis*, might be involved in the telomerase-independent pathway called as alternative lengthening of telomeres [54,55]; target of *osa-MIR2122-p3_1ss19GT* was related to ATM. ATM contributed to the formation of phosphorylated H2AX (γ-H2AX) [56], which is a meiosis-specific isoform of histone H2A (The histone H2A protein encoded by HTA3). Phosphorylated H2AX accumulated at sites of DNA damage when DNA double-strand breaks (DSBs) happened. Targets predicted by *osa-MIR6250-p5* were related to both HTA3 and ATM. Moreover, RAD50 (DNA repair-recombination protein), a component of Mre11/Rad50/Xrs2 (MRX) complex, is essential for meiosis [57,58]. The down-regulation of RAD50 was observed in polyploid rice hybrids during meiosis [15]; the targets predicted by *osa-miR1432-5p_R+1* and *osa-miR5788* were associated with RAD50 in our study. We speculated that these miRNAs (especially the up-regulated miRNAs) might be involved in the interaction network of meiosis-related genes and accompanied with the abnormal regulation of autotetraploid rice, particularly in meiosis.

*osa-miR1432-5p* predicted to target the genes of metal cation transporter (*LOC_Os05g07210*), calcium-transporting ATPase (*LOC_Os04g51610*) and *EF-hand* family (*LOC_Os03g59790*, *LOC_Os03g59870*, *LOC_Os03g59770*), and was found to be up-regulated in the early uninucleate stage of developing pollens of MxA (sterile line) which impair the Ca^2+^-mediated signaling pathway during the rice anther development [59]. In our study, *osa-miR1432-5p_R+1* was significantly up-regulated in autotetraploid rice from PMA to SCP and confirmed by the qRT-PCR, and showed the negative correlation with the target genes in SCP. These results displayed that over expression of *osa-miR1432* might interrupt the Ca^2+^ balance in the PMC and played the crucial roles in autotetraploid rice, so we speculated that this miRNA influence tetraploid pollen viability. Another important miRNA, *osa-miR528*, might be involved in the pollen sterility by targeting the F-box proteins, was also reported by Yan *et al.* [59]. Notably, a negative tendency was observed in SCP between the levels of *osa-miR528-5p* and its target *LOC_Os06g06050* (*F-box* protein) in Taichung65-4x and Taichung65-2x. Further, we also found another miRNA, *osa-miR172d-5p_R+1*, which targeted the *LOC_Os06g06050*. In the present study, *osa-miR172d-5p_R+1* and *osa-miR528-5p* were abundantly expressed in diploid and autotetraploid rice, respectively. The expression of *osa-miR172d-5p_R+1* showed the rising trends gradually from PMA to SCP, while *osa-miR528-5p* showed the contrary trends over the same period. We speculated that *osa-miR528-5p* was dominant in the early stage and then taken over by *osa-miR172d-5p_R+1* in the later period, which silenced the expression of target genes, and finally resulted in abnormal pollen development in autotetraploid rice. However, the relationship between *osa-miR528* and *osa-miR172d-5p_R+1* during the rice reproductive development had not been reported, and this required further study.

### 3.3. Abundance of siRNAs Associated with Transposable Elements May Cause Meiosis Abnormalities in Autotetraploid Rice

Small RNAs are known to be a key component of a signaling network that mediated through DNA methylation and histone modifications [37,60,61,62,63]. Previous study has shown that variation in DNA methylation and activity of class II transposable elements (TEs) play crucial role in the polyploidization of rice to adapt the whole-genome duplication (WGD) [38]. In our study, we abundantly found 24 nt siRNAs associated with both of class I and class II transposable elements, especially Ty3-gypsy in class I and En/Spm type in Class II, in autotetraploid rice during pollen development. Significant decline in 24 nt siRNA TEs associated with developing pollen of diploid rice reflected the activation of gene expression throughout the pollen development and resulted in high seed set. However, 24 nt siRNA maintained its abundance during pollen development in polyploid rice and cause remarkable changes in meiosis compared to diploid rice, suggesting that significant variations during meiosis have a strong influence on the pollen development in polyploid rice. These results are consistent with the previous study, which also found that high chromosomal abnormalities were associated with the altered expression profiles of meiosis related genes in autotetraploid rice [14]. Some 24 nt miRNAs can lead to cytosine DNA methylation at their own loci and target genes, and lead to silencing of gene transcription [64]. *LOC_Os10g26430* has hypermethylation of CHG, not only surrounded by DMRs in its flanking 4-kb regions but also in the gene bodies of autotetraploid rice compared with diploid rice [38]. In our study, we found three DEM, especially *osa-miR5796* that was down-regulated in MA and SCP of Taichung65-4x and predicted a *LOC_Os10g26430* gene, may be associated with the DNA methylation and transposon activity in polyploidization of rice. Taken together, our findings suggest that abundance of siRNAs associated with the transposable elements that involved in DNA methylation in autotetraploid rice, probably influenced the autotetraploid pollen fertility during meiosis.

## 4. Experimental Section

### 4.1. Rice Materials

Autotetraploid rice, Taichung65-4x, and its diploid counterpart, Taichung65-2x, were used in the present study. Taichung65-2x was treated with colchicine to develop Taichung65-4x and self-crossed for more than 22 generations in our lab [14]. They were planted at the experimental site of South China Agricultural University (SCAU) under field conditions. Anthers during three stages of pollen development, including pre-meiotic interphase (PMA), meiosis (MA) and single microspore stage (SCP), were collected from Taichung65-4x and Taichung65-2x as described previously [14]. All samples were stored at −80 °C for RNA extraction.

### 4.2. Small RNA Library Construction, Sequencing and Data Processing

Total RNA was isolated from the anthers using Trizol reagent (Invitrogen, Carlsbad, CA, USA) following the manufacturer’s protocol. The total RNA purity and quantity were analyzed by RNA 6000 Nano LabChip Kit (Agilent, Palo Alto, CA, USA) and Bioanalyzer 2100 with RIN number >7.0. About 1 µg of RNA was used to construct small RNA library according to the protocol of Illumina’s TruSeq small RNA sample preparation Kits (San Diego, CA, USA). Then we executed the single-end sequencing (36 bp) on an Illumina Hiseq2500 at the LC-BIO (Hangzhou, China) according to the manufacturer’s protocol.

After the Illumina sequencing, the raw reads were exposed to the Illumina pipeline filter (Solexa 0.3). To remove common RNA families (tRNA, rRNA, snRNA, snoRNA), adapter dimers, low complexity, junk and repeats, the data were further processed with an in-house program, ACGT101-miR (LC Sciences, Houston, TX, USA) [65]. Consequently, the 18–25 nt length unique sequences were BLASTed to rice precursors in miRBase 20.0 [66] to detect known miRNAs. One mismatch inside of the sequence and length variation at both 3′ and 5′ ends were allowed in the alignment. The unique sequences were mapped to rice mature miRNAs in hairpin arms recognized as known miRNAs, and mapped to the other arm of known rice precursor hairpin opposite to the annotated mature miRNA-containing arm considered to be novel 5p- or 3p-derived miRNAs. The remaining sequences were mapped to other plant species in miRBase 20.0 by BLAST search, and the mapped pre-miRNAs were further BLASTed against the rice genomes [67] to identify their genomic positions. The aforementioned miRNAs were considered as known miRNAs. To identify the novel predicted miRNAs, the unmapped sequences were BLASTed against the rice genome database, and the hairpin RNA structures comprising sequences were predicated by using RNAfold software [68].

Data normalization followed the procedures as described in a previous study [69] with minor modification. (1) Find a common set of sequences among all samples; (2) Construct a reference data set. Each data in the reference set is the copy number median value of a corresponding common sequence of all samples; (3) Perform 2-based logarithm transformation on copy numbers (Log_2_ (copy#)) of all samples and reference data set; (4) Calculate the Log_2_ (copy#) difference (ΔLog_2_ (copy#)) between individual sample and the reference data set; (5) Form a subset of sequences by selecting |ΔLog_2_ (copy#)| < 2, which means less than 4 (2^2^) fold change from the reference set; (6) Perform linear regressions between individual samples and the reference set on the subset sequences to derive linear equations *y* = a*_i_x* + b*_i_*, where a*_i_* and b*_i_* are the slop and interception, respectively, of the derived line, *x* is Log_2_ (copy#) of the reference set, and *y* is the expected Log_2_ (copy#) of sample *i* on a corresponding sequence; (7) Calculate the mid value *x*_mid_ = (max(*x*) − min(*x*))/2 of the reference set. Calculate the corresponding expected Log_2_ (copy#) of sample *i*, *y_i_*_,mid_ = a*_i_*x_mid_ + b*_i_*. Let *y_r_*_,mid_ = *x*_mid_, let Δ*y_i_* = *y_r_*_,mid_ − *y_i_*_,mid_, which is the logarithmic correction factor of sample *i*. We then derive the arithmetic correction factor *fi* = 2^Δ*yi*^ sample *I*; (8) Correct copy numbers of individual samples by multiplying corresponding arithmetic correction factor to original copy numbers.

To find out the siRNAs associated with the transposable elements, we filtered reads that did not match miRNA, rRNA, tRNA, snRNA, or snoRNA, and mapped them against the rice reference genome to select the genome sequences annotated as transposable elements [38]. These subsequent of siRNAs associated with transposable elements (TEs) were used for further analysis. The type of transposable elements (TEs) associated with siRNAs was classified firstly, and then we averaged the expression levels of siRNAs associated with transposable elements in each type to estimate the relative expression levels. These relative expression levels represented the TEs expression levels of each type during each stage in diploid and autotetraploid rice. Small RNA sequencing data have been deposited to NCBI GEO database (Accession Number: GSE79344).

### 4.3. Analysis of Differentially Expressed miRNAs

MicroRNAs were regarded as differentially expressed based on normalized deep-sequencing levels (with the exclusion of 10 RPM) in Taichung65-2X and Taichung65-4X during pollen development. *p*-Value was estimated by selectively by using Chi-square (*X*^2^) test and Fisher exact test. A significance threshold level was set to be 0.05 in each test. miRNAs with *p-*value <0.05 and log_2_ (fold change ratio) >1 were considered as differentially expressed miRNA (DEM). The normalized read count of some miRNAs were set to be 0.01 for further calculation if it has no reads in the library.

### 4.4. The Prediction and Functional Analysis of Target Genes of miRNAs

To predict the genes targeted by DEM, computational target prediction algorithms and TargetFinder [70] were used to detect miRNA binding sites. The algorithm of TargetFinder was based on miRNA and mRNA complementary pairing principle. The miRNA sequence (5′–3′) from 2 to 13 nt was defined as the seed sequence. The mismatching of Guanine and Uracil will lose 0.5 point and the rest of the mismatches cut off 1 point outside the seed sequence, while doubling the lost point in seed sequence. Finally, the results were obtained with the points less than or equal to 4. GO enrichment analysis was done by using AgriGO [71]. Protein–protein interaction networks were used to predict the meiosis-related targets/genes by using STRING v10 [72].

### 4.5. Quantitative Real-Time PCR (qRT-PCR) Analysis

The RNA was extracted from anthers of Taichung65-2x and Taichung65-4x at each stage of pollen development, and used as template for reverse transcription with miRNA-specific stem-loop RT primers [73] using the Transcriptor First Strand cDNA Synthesis Kit (Roche, Mannheim, Germany). The reactions were incubated at 16 °C for 30 min, then pulsed RT of 60 cycles at 30 °C for 30 s, 42 °C for 30 s and 50 °C for 1 s, and a final incubation for 5 min at 85 °C to inactivate the reverse transcriptase [74]. The cDNA template for the miRNA target gene was reverse transcribed using the OligodT20 primer with the Transcriptor First Strand cDNA Synthesis Kit (Roche, Mannheim, Germany).

The qRT-PCRs were performed on Lightcycler480 (Roche) using the SsoAdvanced universal SYBR Green Supermix (Bio-RAD, Hercules, CA, USA). The reaction profile was as follow: 30 s at 95 °C, 40 cycles of 95 °C denaturation for 5 s and 58 °C annealing and extension for 20 s. We performed three biological replications of each reaction, and *U6* snRNA and *ubiquitin* were used as internal control genes for qRT-PCR of DEM and target genes, respectively. The relative expression values of DEM or target genes were calculated based on the *2*^−^^ΔΔ^*^C^*^t^ method [75]. The stem-loop RT primers and target gene-specific primers were designed by using the Primer Premier 5.0 software (Palo Alto, CA, USA) (Table S19).

## Figures and Tables

**Table 1:** Differentially expressed miRNAs (DEM) in autotetraploid rice comparative to diploid rice.

Material	Description/Stage-Specific Expression	Up-Regulated	Down-Regulated	Total
Taichung65-2x	DEM in MA compared to PMA	20	46	66
	DEM in SCP compared to MA	28	96	124
Taichung65-4x	DEM in MA compared to PMA	41	75	116
	DEM in SCP compared to MA	58	57	115
Taichung65-4x *vs.* Taichung65-2x	DEM in PMA	27	19	46
	DEM in MA	37	21	58
	DEM in SCP	75	27	102
	DEM specifically in MA compared to PMA of Taichung65-2x	15	34	49
	DEM specifically in SCP compared to MA of Taichung65-2x	19	55	74
	DEM specifically in MA compared to PMA of Taichung65-4x	36	63	99
	DEM specifically in SCP compared to MA of Taichung65-4x	49	16	65

PMA, MA and SCP represent pre-meiotic interphase, meiosis and single microspore stage, respectively.

**Table 2:** Association between differentially expressed miRNAs (DEM) and meiosis-related genes in autotetraploid rice during pollen development.

miRNA	Targets	Genes Annotation	PMA	MA	SCP	Specific in MA	Meiosis-Related Genes
*osa-miR1320-5p_L-1R+1*	*LOC_Os03g12140*	glucan endo-1,3-β-glucosidase precursor, putative, expressed	up	up	up		*LOC_Os11g03430* (CDC45)
*osa-miR1432-5p_R+1*	*LOC_Os04g51610*	calcium-transporting ATPase, plasma membrane-type, putative, expressed	up	up	up		*LOC_Os04g40290* (CRC1)
	*LOC_Os05g07210*	metal cation transporter, putative, expressed	up	up	up		*LOC_Os02g29464* (RAD50)
*osa-miR1876*	*LOC_Os08g42930*	disease resistance protein RGA1, putative, expressed	ns	up	up		*LOC_Os01g01689* (ATM)
*osa-MIR2122-p3_1ss19GT*	*LOC_Os01g72390*	NBS type disease resistance protein, putative, expressed	ns	up	ns	*osa-MIR2122-p3_1ss19GT*	*LOC_Os01g01689* (ATM)
*osa-miR5788*	*LOC_Os03g07430*	protein kinase domain containing protein, expressed	ns	up	ns	*osa-miR5788*	*LOC_Os12g38460* (MEL2)
	*LOC_Os03g23935*	WD domain, G-β repeat domain containing protein, expressed	ns	up	ns	*osa-miR5788*	*LOC_Os02g40450* (OsMER3)
	*LOC_Os03g53750*	nuclear prelamin A recognition factor, putative, expressed	ns	up	ns	*osa-miR5788*	*LOC_Os06g41050* (OsCOM1)
	*LOC_Os05g39540*	metal cation transporter, putative, expressed	ns	up	ns	*osa-miR5788*	*LOC_Os02g29464* (RAD50)
	*LOC_Os09g09220*	protein kinase domain containing protein, expressed	ns	up	ns	*osa-miR5788*	*LOC_Os12g38460* (MEL2)
*osa-MIR6250-p5*	*LOC_Os01g22500*	craniofacial development protein 1, putative, expressed	ns	up	up		*LOC_Os12g34510* (HTA3)
	*LOC_Os03g63240*	disease resistance protein, putative, expressed	ns	up	up		*LOC_Os01g01689* (ATM)
	*LOC_Os08g39000*	expressed protein	ns	up	up		*LOC_Os03g44760* (OsAM1)
*osa-miR812g*	*LOC_Os11g25510*	protein kinase domain containing protein, expressed	ns	up	up		*LOC_Os12g38460* (MEL2)
*PC-3p-403976_40*	*LOC_Os07g28090*	ABC transporter, ATP-binding protein, putative, expressed	ns	up	up		*LOC_Os02g53680* (OsRPA1A)
*PC-3p-550716_26*	*LOC_Os11g13410*	mla1, putative, expressed	ns	up	ns	*PC-3p-550716_26*	*LOC_Os01g01689* (ATM)
*osa-miR160a-5p_R-1_1ss20CT*	*LOC_Os05g30820*	protein kinase, putative, expressed	ns	down	ns	*osa-miR160a-5p_R-1_1ss20CT*	*LOC_Os12g38460* (MEL2)
*osa-miR167a-3p_L+1R-1*	*LOC_Os03g58260*	indole-3-glycerol phosphate lyase, chloroplast precursor, putative, expressed	ns	down	down		*LOC_Os03g44760* (OsAM1)
	*LOC_Os07g17220*	disease resistance protein, putative, expressed	ns	down	down		*LOC_Os01g01689* (ATM)
*osa-MIR5486-p3*	*LOC_Os01g47256*	exosome complex exonuclease RRP40, putative, expressed	ns	down	down		*LOC_Os01g72880* (MLH1)
	*LOC_Os09g02700*	polyadenylate-binding protein, putative, expressed	ns	down	down		*LOC_Os03g12414* (OsSDS)
	*LOC_Os11g39330*	NB-ARC domain containing protein, putative, expressed	ns	down	down		*LOC_Os01g01689* (ATM)
*osa-MIR5491-p5*	*LOC_Os01g24060*	importin subunit α, putative, expressed	ns	down	ns	*osa-MIR5491-p5*	*LOC_Os03g12414* (OsSDS)
*osa-MIR5518-p3*	*LOC_Os04g48770*	ubiquitin fusion degradation protein, putative, expressed	ns	down	ns	*osa-MIR5518-p3*	*LOC_Os04g40290* (CRC1)
*osa-MIR5806-p5*	*LOC_Os08g19694*	NB-ARC domain containing protein, expressed	ns	down	ns	*osa-MIR5806-p5*	*LOC_Os01g01689* (ATM)
*PC-3p-120829_194*	*LOC_Os03g04520*	RNA recognition motif containing protein, putative, expressed	ns	down	ns	*PC-3p-120829_194*	*LOC_Os02g40450* (OsMER3)

ns: non-significant; Up: up-regulated in autotetraploid rice; Down: down-regulated in autotetraploid rice; PMA, MA and SCP represent pre-meiotic interphase, meiosis and single microspore stage, respectively.

**Table 3:** The regulation of *miR2118* and *miR2275* families in autotetraploid rice.

MicroRNA_Family	miRNA	Sequence (5’ to 3’)	PMA	MA	SCP
*miR2118*	*osa-MIR2118k-p5*	TAAGCTCTATTGTCCCCTCTA	ns	ns	down
	*osa-miR2118e*	TTCCCAATGCCTCCCATGCCTA	ns	ns	up
	*osa-MIR2118l-p5*	TTAGGAAGAGGAAGAAATTGA	ns	down	ns
	*osa-MIR2118m-p5*	GGAATGGGAACATGAAGGAAAG	ns	down	ns
	*osa-MIR2118o-p5*	GGCATGGGGACATGAAGGAATG	ns	down	ns
	*osa-miR2118a*	TTCTCGATGCCTCCCATTCCTA	down	ns	ns
	*osa-miR2118c*	TTCCCGATGCCTCCTATTCCTA	down	ns	ns
	*osa-MIR2118a-p5*	GGACTGGGAACATATGAGAAAG	down	ns	ns
*miR2275*	*osa-miR2275d*	CTTGTTTTTCTCCAATATCTCA	down	ns	up
	*osa-MIR2275c-p3*	ATTGTTTTTCTCCAATATCTCA	down	ns	up
	*mes-miR2275*	TTTGGTTTCCTCCAATATCTTA	down	ns	ns
	*zma-miR2275b-3p_1ss22AG*	TTCAGTTTCCTCTAATATCTCG	down	ns	ns

ns: non-significant; Up: up-regulated in autotetraploid rice; Down: down-regulated in autotetraploid rice; PMA, MA and SCP represent pre-meiotic interphase, meiosis and single microspore stage, respectively.

## Supplementary Materials

Supplementary materials can be found at http://www.mdpi.com/1422-0067/17/4/499/s1.
